# Complete response in metastatic clear cell renal cell carcinoma following pembrolizumab–axitinib and subsequent nephrectomy: a Case Report

**DOI:** 10.3389/fimmu.2026.1811163

**Published:** 2026-05-01

**Authors:** Mariam Grazia Polito, Davide Caruso, Antonella Cosimati, Carla Adriana Ramirez Reinaga, Etien Leka, Andrea Fuschi, Antonio Luigi Pastore, Antonio Carbone, Kostika Lako, Claudio Di Cristofano, Vincenzo Petrozza, Daniele Santini, Gian Paolo Spinelli

**Affiliations:** 1 UOC Oncologia Territoriale – ASL Latina – CDS Aprilia, La Sapienza Università Di Roma Polo Pontino, Latina, Italy; 2 Department of Urology Università “La Sapienza”, ICOT Polo Pontino, Latina, Italy; 3 Department of Medical and Surgical Science and Biotechnology, Università “La Sapienza”, Rome, Italy; 4 Department of Medico-Surgical Sciences and Biotechnologies Histopathology, Sapienza University of Rome Faculty of Pharmacy and Medicine, Latina, Italy

**Keywords:** complete response, immune checkpoint inhibitor, nephrectomy, renal cell adenocarcinoma, TKI - tyrosine kinase inhibitor

## Abstract

**Background:**

Renal cell carcinoma (RCC) is the most common kidney cancer in adults. The advent of immune checkpoint inhibitors (ICIs), particularly in combination with tyrosine kinase inhibitors (TKIs), has significantly improved outcomes in advanced disease.

**Case presentation:**

A 67-year-old woman with stage IV clear cell RCC and intermediate-risk features presented with pulmonary, nodal, and peritoneal metastases. She received first-line pembrolizumab plus axitinib, achieving durable disease control and partial response with good tolerability. After 32 months she achieved sustained disease stability and underwent robot-assisted left nephrectomy. Histology revealed complete pathological response. Postoperatively, pembrolizumab was continued with a pseudo-adjuvant intent, and as of March 2026, the patient remains in radiological near-complete response with a progression-free survival of approximately 41 months from treatment initiation.

**Discussion and conclusions:**

This case illustrates that ICI–TKI combinations can induce profound and durable responses even in intermediate-risk metastatic RCC. Consolidative surgery after systemic control, though still under debate, may be beneficial for carefully selected patients. Overall, this report underscores the potential of immunotherapy–TKI combinations to achieve long-term remission and the importance of a multidisciplinary, personalized treatment approach in advanced RCC.

## Introduction

Renal cell carcinoma (RCC) represents the most common type of kidney cancer in adults, with clear cell histology being the predominant subtype. Historically, patients with intermediate-risk disease faced a poor prognosis, with median overall survival (mOS) ranging from 6.9 to 18.4 months depending on the conventional therapies available. The introduction of immune checkpoint inhibitors (ICIs), particularly agents targeting programmed cell death protein-1 (PD-1), in combination with vascular endothelial growth factor receptor (VEGFR) tyrosine kinase inhibitors (TKIs), has revolutionized first-line treatment, profoundly improving both response rates and overall survival (1). In recent years, combination strategies have demonstrated durable responses and long-term disease control, reshaping the therapeutic landscape of advanced RCC.

In particular, the phase 3 randomized KEYNOTE-426 trial demonstrated that the combination of the anti–PD-1 antibody pembrolizumab plus the VEGFR TKI axitinib improved progression-free survival (PFS) compared with sunitinib, with a median of 15.1 vs. 11.1 months (hazard ratio (HR) 0.69; 95% CI: 0.57–0.84; p < 0.001). This benefit also translated into overall survival (OS), with 60-month OS rates of 41.9% versus 37.1% for the combination and sunitinib, respectively. Median duration of response was 23.6 months for pembrolizumab plus axitinib. In subgroup analyses, the benefit was greater for patients with intermediate- and poor-risk disease according to International Metastatic Renal Cell Carcinoma Database Consortium (IMDC) criteria. Although partial responses are common, complete responses (CR) remain relatively rare, occurring in approximately 5%, underscoring the clinical significance of exceptional responders (2–4).

Other PD-1 + VEGFR TKI combinations, such as lenvatinib–pembrolizumab and avelumab–axitinib, have demonstrated efficacy in advanced RCC, but for the purposes of this case, the discussion focuses on pembrolizumab plus axitinib, the regimen administered to the patient (5, 6).

Rates of CR with ICI–TKI therapy highlight the potential for profound disease control, but also underscore the need to identify patients who may achieve exceptional responses. In this context, the role of deferred or consolidative cytoreductive nephrectomy has emerged as an important consideration. Although historically cytoreductive nephrectomy was performed upfront, recent evidence suggests that in patients achieving sustained systemic responses with ICI–TKI combinations, delayed nephrectomy may provide additional benefit, potentially improving long-term outcomes, although randomized data are still pending (e.g., PROBE trial) (7).

The biologic plausibility of achieving a pathologic complete response is supported by the robust immune activation induced by PD-1 blockade combined with VEGFR inhibition, which can promote tumor necrosis, lympho-histiocytic infiltration, and the elimination of residual tumor cells. Understanding these dynamics is critical to contextualize rare but clinically meaningful outcomes, such as the complete pathological response observed in this case.

Given the encouraging results of ICIs in various settings, identifying predictive biomarkers of response to immunotherapy in these patients is of great interest, as it could help select those most likely to benefit and optimize the duration of treatment. Predictive biomarkers, such as pre-treatment neutrophil-to-lymphocyte ratio (NLR), have been associated with response to ICIs targeting the PD-1/PD-L1 axis, with NLR less than 3 correlating with longer PFS and OS in retrospective studies. Although other exploratory biomarkers have been investigated, their clinical utility remains uncertain and they are not central to the current clinical scenario (10).

## Case presentation

A 67-year-old female presented to our clinic in May 2022 after an incidental detection of a renal mass. She had no significant comorbidities, was a non-smoker, and reported no family history of cancer. Her initial complaint was lumbosacral pain, leading to a lumbar spine MRI, which unexpectedly revealed an expansive lesion in the left kidney measuring approximately 8 cm with both solid and partially cystic components. Routine hematological investigations revealed no significant abnormalities. The basal neutrophil-to-lymphocyte ratio (NLR) was 2.7.

**Figure 1:**
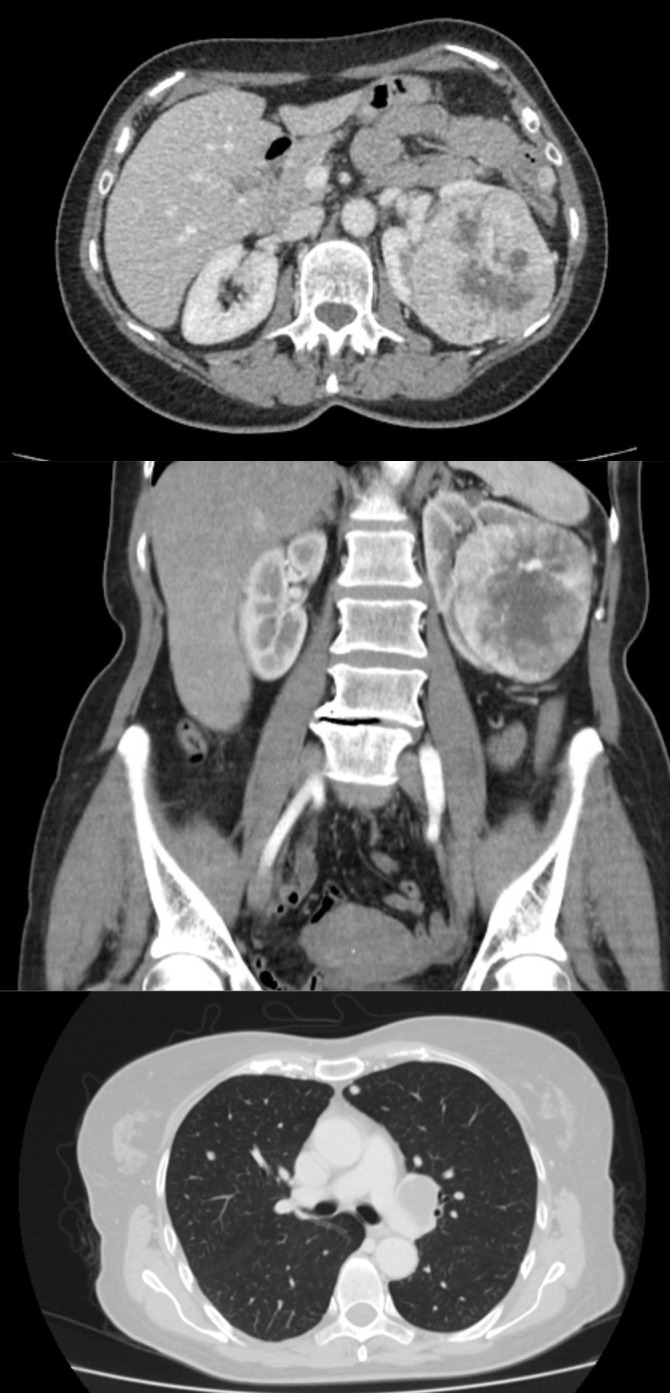
Baseline CT images showing the left renal primary tumor, a largely necrotic 9 × 7 cm mass, in both axial (top) and coronal (middle) planes, along with a peritoneal metastasis in the left paracolic gutter and bilateral pulmonary metastases (axial, bottom).

A subsequent contrast-enhanced whole-body CT scan revealed multiple bilateral pulmonary nodules, up to 10 mm in diameter, and nodal involvement at the left pulmonary hilum. The left renal fossa was occupied by a largely necrotic 9 × 7 cm mass, infiltrating the renal capsule and extending toward pelvic structures, without evidence of renal vein thrombosis. Additionally, two pathological nodules, measuring up to 13 mm, were identified in the left paracolic gutter (**Figure 1**).

**Figure 2:**
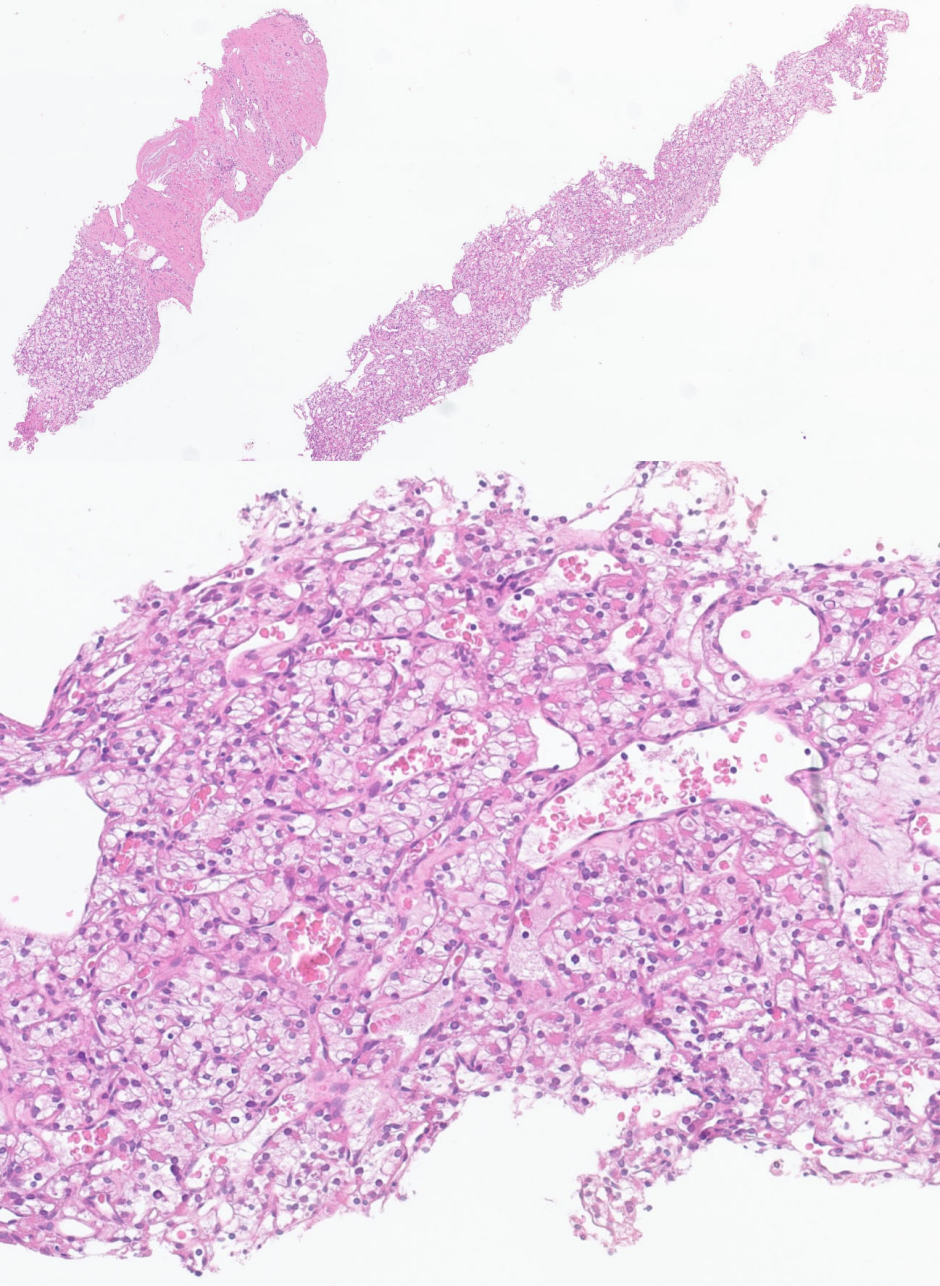
Previous renal core needle biopsy showing clear cell renal cell carcinoma (H.E, 2x, top; 10x, bottom). At higher magnification, note the nested architecture and prominent network of thin-walled vessels, consistent with ISUP grade 1.

A renal biopsy was performed on June 26, 2022 without complications. Histopathological analysis confirmed clear cell carcinoma, grade 1 (**Figure 2**) (PD-L1 testing was not performed at baseline). According to International Metastatic Renal Cell Carcinoma Database Consortium (IMDC) criteria, the patient was classified as intermediate risk, meeting one criterion: time from diagnosis to systemic therapy < 1 year.

Following multidisciplinary discussion, first-line systemic therapy with the anti–PD-1 antibody Pembrolizumab in combination with the vascular endothelial growth factor receptor (VEGFR) tyrosine kinase inhibitor (TKI) Axitinib, administered every 21 days, was recommended.

Prior to initiating pembrolizumab, baseline morning cortisol was measured as part of routine endocrine assessment at our institution for patients considered for immune checkpoint inhibitor therapy. The patient exhibited mildly elevated morning cortisol. ACTH levels were within the normal range, and no clinical features suggestive of Cushing syndrome were present. Given these findings, pembrolizumab initiation was temporarily withheld to allow for endocrinologic evaluation and to rule out occult adrenal pathology. After a full assessment, including repeat morning cortisol measurements, the endocrinology team concluded that the elevation was transient and not indicative of active hypercortisolism or adrenal disease. First-line therapy was initiated with axitinib 5 mg orally twice daily starting in July 2022, while the endocrinology evaluation was ongoing. Pembrolizumab was subsequently introduced at a dose of 200 mg intravenously every 21 days starting on August 19, 2022, completing the planned combination regimen.

The therapy was well tolerated. Throughout the approximately 32-month course of combination therapy prior to surgery, the patient underwent routine laboratory monitoring every 3–4 weeks, including complete blood counts, liver and renal function tests, thyroid function (TSH, free T4), and cortisol levels. No grade ≥3 immune-related adverse events were observed. Minor events included grade 1 fatigue and grade 2 hypertension, managed conservatively. Liver and kidney function remained within normal limits, and thyroid function was stable without intervention.

In the meantime, the patient continued regular endocrinological monitoring.

After four cycles, a CT reassessment in November 2022 demonstrated reduction of the bilateral pulmonary nodules, reduction of the left renal lesion to 37 × 35 mm, and disappearance of the previously noted paracolic nodules. Given the partial response and good tolerability, the patient continued therapy, undergoing regular CT scans every four months, all of which confirmed sustained disease stability.

**Figure 3:**
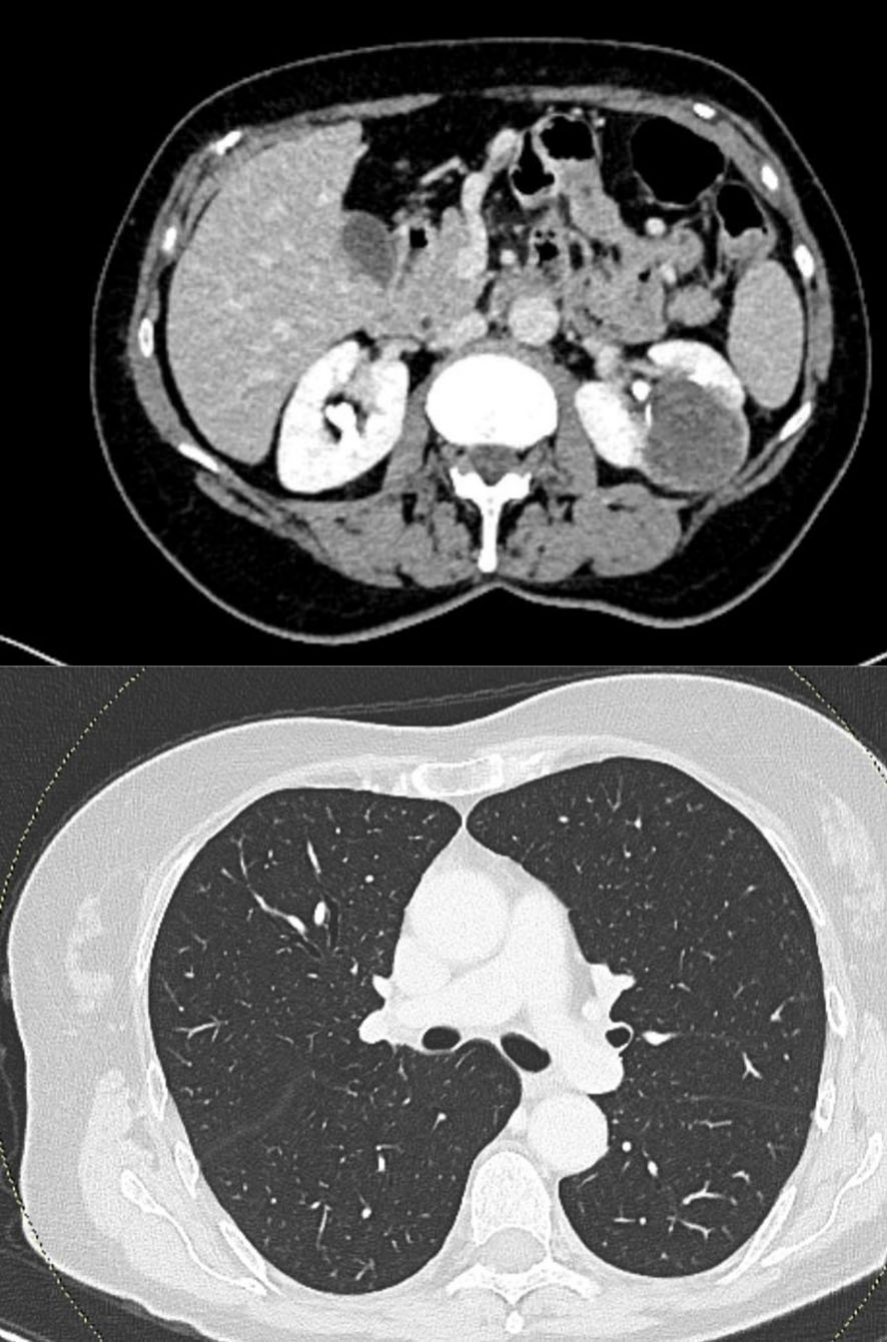
CT scan after four cycles of therapy showing reduction of the left renal lesion to 37 × 35 mm (top), disappearance of the previously noted paracolic nodules (top), and reduction of bilateral pulmonary nodules (bottom).

By February 2025, imaging showed stability of the primary renal lesion, persistent millimetric pulmonary nodules, and disappearance of the peritoneal nodules (**Figure 3**).

Given this prolonged radiological stability, the case was reviewed in a multidisciplinary setting, and surgical intervention was recommended. The decision for consolidative nephrectomy was based on multidisciplinary assessment, taking into account: sustained reduction of the primary renal lesion to 37 × 35 mm, disappearance of peritoneal nodules, stability of residual millimetric pulmonary nodules, absence of new metastatic lesions over approximately 32 months of systemic therapy, and the patient’s excellent tolerance to treatment.

Given the lack of a universally established standard for the optimal duration of immunotherapy in the metastatic setting, the decision to continue pembrolizumab beyond the conventional duration was made in a multidisciplinary context, with treatment individualized based on sustained clinical benefit, excellent tolerability, and the patient’s strong motivation, despite a duration of approximately two years generally being adopted in clinical practice.

**Figure 4:**
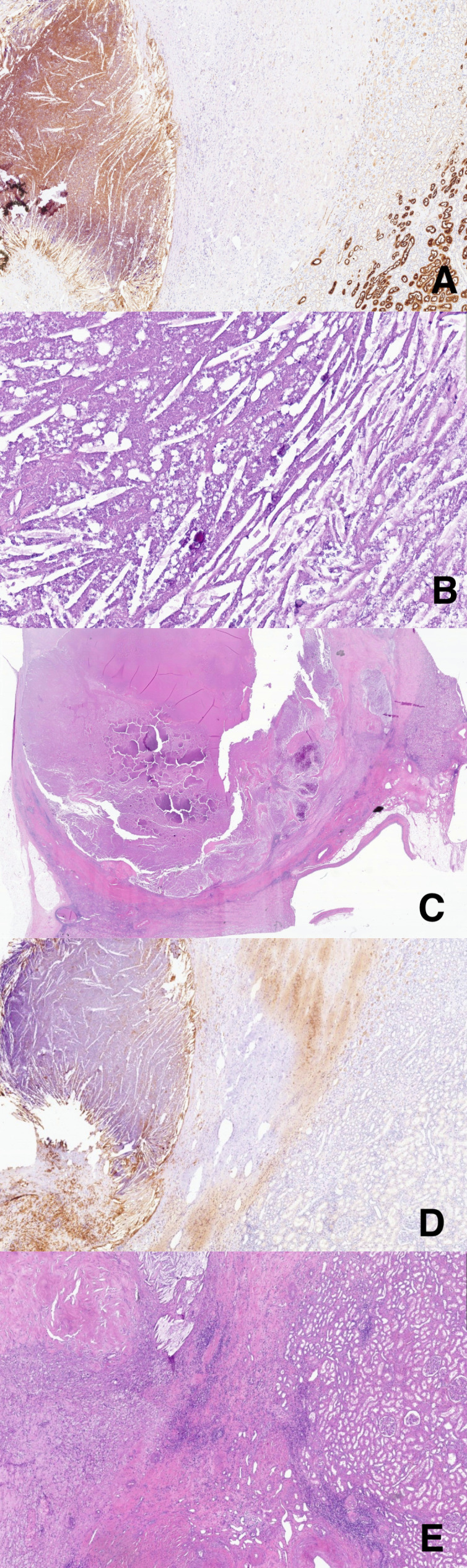
Histological images from the radical nephrectomy specimen. At low magnification, **(A)** (CD10 5x) shows CD10-positive renal tubules, while **(C)** (H.E 2x) demonstrates the necrotic tumor bed. At higher magnification, **(E)** (H.E 20x) and **(B)** (H.E 40x) confirm extensive necrosis with dystrophic calcifications and cholesterol clefts, without evidence of viable tumor cells. **(D)** (CD68 20x) highlights numerous CD68-positive histiocytes at the interface between necrotic tissue (former tumor) and residual renal parenchyma.

Consequently, after 43 cycles of pembrolizumab and axitinib, the patient underwent a robot-assisted left nephrectomy in March 2025, which was completed without complications. Histological examination revealed a fibro-necrotic nodule with features of chronic lympho-histiocytic inflammation, consistent with a complete response (**Figure 4**).

Following surgery, the patient was again evaluated in a multidisciplinary setting. Considering both the histological findings and imaging review, revealing near-complete response, she was started on pseudo-adjuvant pembrolizumab with the intention to complete a total of one year of postoperative therapy. By March 2026, she had received her 13th postoperative cycle. She tolerated the treatment well and will continue in the absence of recurrence, until completion of one year of treatment.

The decision reflected a pragmatic clinical approach supported by indirect evidence from trials investigating perioperative immunotherapy and by expert consensus within the multidisciplinary team.

The persistent millimetric pulmonary nodules observed postoperatively are most likely to represent fibrosis or post-treatment scarring rather than residual viable tumor. Overall, the clinical and pathological findings are consistent with a near-complete response, further supported by the complete pathological response observed in the nephrectomy specimen and long-term radiological stability on follow-up imaging. Continued monitoring is warranted.

As of the latest CT scan in March 2026, the patient has maintained a progression-free survival of approximately 41 months from the initiation of pembrolizumab plus axitinib, remaining in radiological near-complete response with preserved quality of life.

The patient’s treatment and clinical course are summarized in **Table 1**.

**Table 1:** Overview of the patient’s therapy, key clinical events, imaging findings and treatment-related adverse events.

Date	Timeline event	Details	Notes
July 2022	Start of Axitinib	Axitinib 5 mg orally twice daily	
August 2022	Initiation of Pembrolizumab	200 mg IV every 3 weeks	Cortisol elevation delayed start; otherwise well tolerated
October 2022	CT scan	First CT scan: Partial response	Grade 1 fatigue, Grade 2 hypertension
March 2025	Surgical Intervention	Cytoreductive nephrectomy	
March 2026	13th postoperative cycle	Pembrolizumab ongoing	Stable, no evidence of disease

## Discussion

In this case, a patient with stage IV clear cell renal cell carcinoma achieved a PFS of approximately 41 months on first-line pembrolizumab plus axitinib, as documented at the last radiological assessment in March 2026. Following consolidative nephrectomy in March 2025, she has maintained progression-free survival, and the most recent CT scan performed in March 2026 confirmed stable findings.

This case is remarkable for several reasons. Despite an intermediate-risk profile according to IMDC criteria, associated with metastatic site generally linked to less favorable prognosis (peritoneal involvement), first-line systemic therapy with the anti–PD-1 antibody pembrolizumab in combination with the VEGFR TKI axitinib induced a durable partial response, eventually leading to complete remission.

The identification of predictive factors for response and response duration to immune checkpoint inhibitor (ICI)-based therapy remains an area of active research.

An additional significant aspect is the role of surgery. The patient underwent resection of the primary renal lesion after achieving long-term systemic disease control. It is important to note that the intermediate-risk group is heterogeneous; patients with a single IMDC risk factor may experience significantly better outcomes than those with two factors. In this case, the patient had only one IMDC risk factor (time from diagnosis to systemic therapy <1 year), consistent with a relatively favorable prognosis within the intermediate-risk category. Updated data from the CARMENA trial suggest that upfront cytoreductive nephrectomy may confer benefit in patients with a single IMDC risk factor (8).

Recent Phase 3 studies, including the PROBE trial (SWOG S1931), are investigating the role of deferred cytoreductive nephrectomy following ICI-based therapy, highlighting the evolving landscape for integrating systemic therapy and surgery (7).

In patients currently awaiting results from emerging therapies, such as HIF-2α inhibitors or other novel agents, consolidative surgery may provide durable disease control, particularly given the limited effective options in subsequent treatment lines and historically modest outcomes (9). However, with the advent of new systemic therapies, the potential benefit of surgery will need to be carefully reassessed in the context of evolving treatment paradigms.

However, the indication for consolidative nephrectomy in patients achieving profound systemic responses remains controversial. In the present case, histology revealed primarily fibro-necrotic tissue with no viable tumor, raising the question of whether surgical intervention materially altered the disease course. While the multidisciplinary team judged the procedure to be potentially beneficial in consolidating remission, it is important to acknowledge potential risks, including perioperative morbidity and long-term impact on renal function, which must be carefully weighed against the uncertain incremental benefit in patients with excellent systemic response.

From an immunological perspective, the histological findings in the surgical bed of fibro-necrotic tissue with chronic lympho-histiocytic inflammation suggest a robust immune infiltration, potentially triggered and maintained by pembrolizumab-axitinib therapy, supporting the concept of immunologically mediated tumor clearance.

Another relevant aspect concerns the optimal duration of systemic therapy. In metastatic renal cell carcinoma, clinical experience and trials evaluating ICI-based combinations have adopted heterogeneous treatment strategies, with some protocols limiting immunotherapy to a fixed duration (e.g., two years), while others allow treatment continuation until disease progression or unacceptable toxicity. In the present case, prolonged treatment with pembrolizumab plus axitinib was associated with durable disease control; however, it remains uncertain whether similar outcomes could have been achieved with shorter treatment exposure. This uncertainty highlights the need for individualized treatment decisions, balancing potential benefits of continued therapy against cumulative toxicity, cost, and patient preference.

Taken together, these observations underscore the importance of a personalized, multidisciplinary approach that integrates systemic therapy with locoregional interventions for patients achieving deep and durable responses, while carefully considering the timing, necessity, and potential risks of surgical intervention, as well as the appropriate duration of systemic therapy.

## Conclusions

This case demonstrates that immunotherapy–TKI combinations can achieve durable disease control in intermediate-risk, stage IV clear cell renal cell carcinoma while preserving the patient’s quality of life. The outcome also highlights a potential role for surgery as a consolidative intervention, after careful multidisciplinary evaluation. However, the exact benefit of consolidative nephrectomy following systemic disease control remains uncertain, emphasizing the need for individualized, multidisciplinary decision-making. Pseudo-adjuvant pembrolizumab offered a rational strategy to maintain disease control, consistent with current evidence and expert consensus.

Overall, this case underscores that a subset of patients with metastatic RCC can achieve profound and durable responses to ICI–TKI combinations, and reinforces the importance of a personalized, multidisciplinary approach that integrates systemic therapy with locoregional interventions, balancing potential benefits against procedural risks and treatment duration considerations.

## Data Availability

The raw data supporting the conclusions of this article will be made available by the authors, without undue reservation.

## References

[1] Nayak D Akers KG Frederickson AM Mbous YPV Aguiar-Ibáñez R . Systematic literature review of real-world evidence on overall survival in cancer patients before and after the approval of anti-PD-(L)1 therapy. Front Oncol. (2025) 15:1615795. doi: 10.3389/fonc.2025.1615795. PMID: 40831930 PMC12358273

[2] Ko JJ Xie W Kroeger N Lee JL Rini BI Knox JJ . The International Metastatic Renal Cell Carcinoma Database Consortium model as a prognostic tool in patients with metastatic renal cell carcinoma previously treated with first-line targeted therapy: a population-based study. Lancet Oncol. (2015) 16:293–300. doi: 10.1016/S1470-2045(14)71222-7, PMID: 25681967

[3] Powles T Plimack ER Soulières D Waddell T Stus V Gafanov R . Pembrolizumab plus axitinib versus sunitinib monotherapy as first-line treatment of advanced renal cell carcinoma (KEYNOTE-426): extended follow-up from a randomized, open-label, phase 3 trial. Lancet Oncol. (2020) 21:1563–73. doi: 10.1016/S1470-2045(20)30436-8, PMID: 33284113

[4] Heng DY Xie W Regan MM Warren MA Golshayan AR Sahi C . Prognostic factors for overall survival in patients with metastatic renal cell carcinoma treated with vascular endothelial growth factor–targeted agents: results from a large, multicenter study. J Clin Oncol. (2009) 27:5794–9. doi: 10.1200/JCO.2008.21.4809, PMID: 19826129

[5] Powles T Burotto M Escudier B Apolo AB Bourlon MT Shah AY . Nivolumab plus cabozantinib versus sunitinib for first-line treatment of advanced renal cell carcinoma: extended follow-up from the phase III randomized CheckMate 9ER trial. ESMO Open. (2024) 9:102994. doi: 10.1016/j.esmoop.2024.102994, PMID: 38642472 PMC11046044

[6] Tannir NM Albigès L McDermott DF Burotto M Choueiri TK Hammers HJ . Nivolumab plus ipilimumab versus sunitinib for first-line treatment of advanced renal cell carcinoma: extended 8-year follow-up results of efficacy and safety from the phase III CheckMate 214 trial. Ann Oncol. (2024) 35:1026–38. doi: 10.1016/j.annonc.2024.07.727, PMID: 39098455 PMC11907766

[7] Jeyakumar G Kim S Bumma N Landry C Silski C Suisham S . SWOG S1931 (PROBE): Phase III randomized trial of immune checkpoint inhibitor (ICI) combination regimen with or without cytoreductive nephrectomy (CN) in advanced renal cancer [NCT04510597. Urol Oncol Semin Orig Investig. (2024) 42:S4. doi: 10.1016/j.urolonc.2024.01.044, PMID: 38826717

[8] Méjean A Ravaud A Thezenas S Colas S Beauval JB Bensalah K . Sunitinib alone or after nephrectomy in metastatic renal-cell carcinoma. N Engl J Med. (2018) 379:417–27. doi: 10.1056/NEJMoa1803675, PMID: 29860937

[9] Choueiri TK Powles T Peltola K de Velasco G Burotto M Suarez C . Belzutifan versus everolimus for advanced renal-cell carcinoma. N Engl J Med. (2024) 391:710–21. doi: 10.1056/NEJMoa2313906, PMID: 39167807

[10] Vaishampayan U Tangen C Tripathi A Shuch B Pal S Barata P . Neutrophil lymphocyte ratio and duration of prior anti-angiogenic therapy as biomarkers in metastatic RCC receiving immune checkpoint inhibitor therapy. J Immunother Cancer. (2017) 5:82. doi: 10.1186/s40425-017-0287-5, PMID: 29041991 PMC5646127

